# Status of and Future Research on Thermosensory Processing

**DOI:** 10.3389/fphys.2016.00150

**Published:** 2016-04-25

**Authors:** Makoto Mizunami, Hiroshi Nishino, Fumio Yokohari

**Affiliations:** ^1^Faculty of Science, Hokkaido UniversitySapporo, Japan; ^2^Research Institute for Electronic Science, Hokkaido UniversitySapporo, Japan; ^3^Department of Earth System Science, Fukuoka UniversityFukuoka, Japan

**Keywords:** cold receptor neurons, warm receptor neurons, antennal lobe, thermosensory projection neurons, lateral protocerebrum, insects

## Abstract

Thermosensation is critically important for survival of all animals. In the cockroach *Periplaneta americana*, thermoreceptor neurons on antennae and thermosensory interneurons in the antennal lobe have been characterized electrophysiologically, and recent studies using advanced transgenic technologies in the fruit fly *Drosophila melanogaster* have added much to the knowledge of these neurons, enabling us to discuss common principles of thermosensory processing systems in insects. Cockroaches and many other insects possess only one type of thermoreceptor neurons on antennae that are excited by cooling and inhibited by warming. In contrast, the antennae of fruit flies and other dipterans possess oppositely responding warm and cold receptor neurons. Despite differences in their thermoreceptive equipment, central processing of temperature information is much the same in flies and cockroaches. Axons of thermoreceptor neurons project to the margin of the antennal lobe and form glomeruli, from which cold, warm and cold-warm projection neurons originate, the last neurons being excited by both cooling and warming. Axons of antennal lobe thermosensory projection neurons of the antennal lobe terminate in three distinct areas of the protocerebrum, the mushroom body, lateral horn and posterior lateral protocerebrum, the last area also receiving termination of hygrosensory projection neurons. Such multiple thermosensory pathways may serve to control multiple forms of thermosensory behavior. Electrophysiological studies on cockroaches and transgenic approaches in flies are encouraged to complement each other for further elucidating general principles of thermosensory processing in the insect brain.

Detection of temperature is one of the most fundamental sensory functions in all species and is critical for animal survival. Insects are particularly useful for the study of thermosensory systems since they are heterothermic and thus have to move to favorable temperature areas by tracing a small thermal gradient in the air. Indeed, studies on insects have contributed to the understanding of cellular and molecular mechanisms of thermoreception (Yokohari, 1999; Barbagallo and Garrity, 2015).

Insects are also suitable animals for the study of thermosensory processing in the brain. Two recent studies have shown thermosensory processing pathways in the brain of the fruit fly *Drosophila melanogaster* (Frank et al., 2015; Liu et al., 2015). In one study, thermosensory projection neurons originating from the posterior antennal lobe, which is the termination area of thermoreceptor neurons on the antennae, were characterized (Frank et al., 2015). The other study focused on integration of thermosensory signals between the receptor neurons and projection neurons (Liu et al., 2015). The major findings in those studies have been concisely reviewed (Florence and Reiser, 2015). Those papers, however, did not refer to the fact that thermoreceptor neurons on antennae (Nishikawa et al., 1992, 1995; Yokohari, 1999) and thermosensory projection neurons in the antennal lobe (Nishikawa et al., 1991; Zeiner and Tichy, 2000; Fisher and Tichy, 2002; Nishino et al., 2003) have been anatomically and physiologically characterized in the cockroach *Periplaneta americana*. In science, results of new studies in one species should be integrated to existing knowledge in another species in order to obtain broader perspectives. The aim of this article is to fill the gap between the present and the past and to provide perspectives for the future. We discuss basic principles of thermosensory processing in the insect brain by integrating findings in the cockroach and the fly.

## Thermoreceptor neurons on the antennae

**Figure 1:**
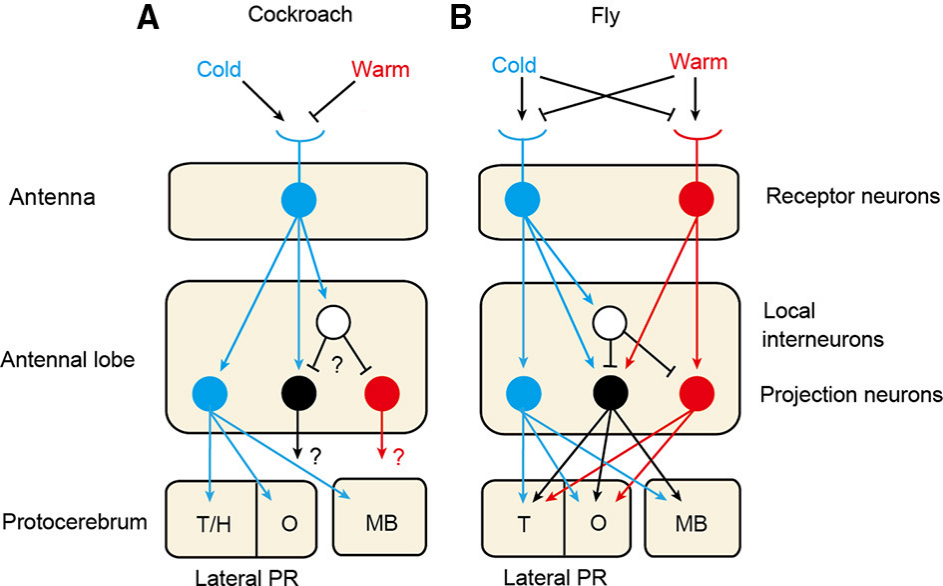
**Thermosensory systems in the cockroach (A) and the fruit fly (B)**. The cockroach is equipped with cold receptor neurons on the antennae (Nishikawa et al., 1992), whereas the fruit fly possesses cold and warm receptors (Gallio et al., 2011). In both species, axons of thermoreceptors terminate in a specific area at the posterior margin of the antennal lobe and form glomerular structures, called cold and warm glomeruli in the fly and cold glomeruli (DC1) in the cockroach (Nishino et al., 2003; Frank et al., 2015). In the cockroach, cold projection neurons originating from the cold glomeruli exhibit excitatory responses to cooling. The cockroach also possesses warm and warm-cold projection neurons that exhibit excitatory responses to warming and to both warming and cooling, respectively, the morphologies of which have not been characterized (Zeiner and Tichy, 2000; Fisher and Tichy, 2002). The fruit fly also possesses cold, warm and warm-cold projection neurons (Frank et al., 2015; Liu et al., 2015). In both species, axons of these neurons project to calyces of the mushroom body (MB) and the olfactory area (O) and non-olfactory area of the lateral protocerebrum (PR) (Frank et al., 2015; Nishino et al., 2003). In the cockroach, cold glomeruli form a cluster with moist and dry glomeruli, the latter two glomeruli receiving termination of moist and dry receptors, respectively (Nishikawa et al., 1995), and projection neurons originating from the cold, moist and dry glomeruli converge to the same olfactory and non-olfactory areas of the lateral PR (Nishino et al., 2003). T/H: thermo- and hygrosensory area; T: thermosensory area of the lateral PR.

Figure 1 schematically illustrates the thermosensory systems of the cockroach and fruit fly. The fruit fly is equipped with highly sensitive cold and warm (or hot) receptors on the antennae (Gallio et al., 2011), as is the mosquito (Davis and Sokolove, 1975), the former being excited by warming and inhibited by cooling, and the latter having the opposite response polarity. On the other hand, the antennae of many other species of insects, including cockroaches, locusts, crickets, moths, honey bees and ants, have only cold receptors that are excited by cooling and inhibited by warming (Yokohari, 1999). In species that possess only one receptor type, cold receptors provide both cooling and warming information necessary to orient toward an appropriate temperature. In species that have two receptor types, integration of signals from the two types of receptors may lead to sophisticated processing of temperature information. It seems that thermoreception by a one-receptor-type system is an evolutionarily ancient type and that warm receptors emerged later during the course of evolution of dipterans, which might have reflected the need for very small animals like flies to rapidly escape from the heat.

There are similarities and differences in the arrangement of thermosensory receptors on antennae between the fly and the cockroach. In the fruit fly, three cold receptor neurons and three warm receptor neurons are housed in internal sensilla of the arista, a large bristle attached to the last segment of the antenna (Foelix et al., 1989; Gallio et al., 2011). In addition, a small number (15–10) of cold receptor neurons are housed in the sacculus of the third segment of the antenna (Gallio et al., 2011). The antennae of the cockroach are equipped with two types of very sensitive cold receptors. One type is housed in sensilla that also contain four types of olfactory receptors (Nishikawa et al., 1992, 1995). The other type is housed in sensilla that also contain two types of hygroreceptors called moist and dry receptors, which are excited by an increase or a decrease in relative humidity (Nishikawa et al., 1992, 1995). Hygroreception is needed for insects to detect water vapor in the environment, which is critical for survival of small animals such as insects (Yokohari, 1999). Recent studies show that the third segment (Liu et al., 2007) and the arista (Ji and Zhu, 2015) of the antennae of the fruit fly contain hygroreceptor neurons.

## Sensory processing in the primary thermosensory center

There is a similarity between the fruit fly and the cockroach in that axons of thermosensory neurons project to specific glomeruli at the margin of the antennal lobe. In the fly, axons of antennal thermosensory neurons project to a specific area at the margin of the antennal lobe, called the posterior antennal lobe (Frank et al., 2015), in which they form two separate glomeruli, called cold and warm glomeruli (Gallio et al., 2011). The fruit fly also possesses internal warm receptors located inside the head capsule (Hamada et al., 2008), and the termination areas of their axons differ from those of thermoreceptors on the antennae: they terminate in VL2a and VL2b glomeruli in the antennal lobe (Hamada et al., 2008) and the superior lateral protocerebrum (Galili et al., 2014). The presence and the central projection of internal thermoreceptors have not been examined in insects other than flies. In the cockroach, axons of cold receptors converge to three fused glomeruli, called DC1 glomeruli (DC indicating dorso-central according to the neuraxis), located at the posterior margin of the antennal lobe (Nishino et al., 2003). Notably, these glomeruli form clusters with two sets of glomeruli that receive axon terminals of hygroreceptor neurons, one set being three fused glomeruli called DC2 and the other being two separate DC3 glomeruli (Nishino et al., 2003). Similarly in the fruit fly, axons of hygroreceptor neurons terminate at the margin of the antennal lobe (Liu et al., 2007).

There are similarities, as well as differences, in sensory processing in the primary thermosensory center between the cockroach and the fly. In the fruit fly, projection neurons originating from cold and warm glomeruli are termed cold and warm projection neurons, respectively (Liu et al., 2015). Electrophysiological studies have suggested that cold projection neurons receive signals from cold receptors, whereas warm projection neurons integrate excitatory signals from warm receptors and inhibitory signals from cold receptors, via local inhibitory interneurons. Integration of the excitatory and inhibitory inputs may provide a sensitive measurement of changes in temperature (Liu et al., 2015). The warm pathway is more elaborated compared to the cold pathway, which may indicate that the warm pathway has been evolved by modification of pre-existing circuitry to process cold receptor signals. In addition to the warm and cold projection neurons, the fruit fly possesses a third type of thermosensory projection neurons that are excited by both warming and cooling (Frank et al., 2015; Liu et al., 2015), and these neurons have been suggested to integrate signals from cold and warm receptors and from inhibitory local interneurons (Liu et al., 2015). Genetically silencing these receptors and projection neurons prevent the fly' avoidance of harmfully cold and hot temperatures (Gallio et al., 2011).

Cockroaches possess cold and warm projection neurons that exhibit responses to a 0.5°C decrease and 0.5°C increase in temperature, respectively (Fisher and Tichy, 2002; Nishino et al., 2003). They also possess warm-cold (ON-OFF) projection neurons, which also respond to some odors and thus are called bimodal neurons (Zeiner and Tichy, 2000; Fisher and Tichy, 2002). Physiological studies have suggested that cold receptor neurons provide excitatory signals to cold and warm-cold projection neurons and that they provide inhibitory signals to warm and warm-cold projection neurons via inhibitory local interneurons (Zeiner and Tichy, 2000; Fisher and Tichy, 2002). Thus, neural pathways from the cold receptors to cold, warm and warm-cold projection neurons appear to be conserved between the fruit fly and the cockroach.

In the cockroach antennal lobe, cold and warm signals (OFF and ON signals) encoded as excitatory and inhibitory processes in cold receptor neurons are separated into different classes of projection neurons, each encoding ON, OFF, and ON-OFF signals as an excitatory process. This reminds us of the vertebrate visual system, in which signals on decrement and increment of light intensity from a background level (OFF and ON signals) are encoded at first as excitatory and inhibitory processes in photoreceptors and then separated into ON, OFF, and ON-OFF ganglion cells, each encoding ON, OFF, and ON-OFF signals as an excitatory process. As a result of this signal conversion, ON and OFF signals are encoded without a high rate of spontaneous spike activity, which is economically advantageous (Shiller et al., 1986). In the fly, separate ON, OFF, and ON-OFF channels are formed by integration of signals from two types of thermoreceptor neurons.

## Thermosensory pathways in the protocerebrum

Similarity in thermosensory processing in flies and cockroaches becomes more obvious when comparing termination areas of thermosensory projection neurons in the protocerebrum. In the fruit fly, axons of thermosensory projection neurons enter the protocerebrum and terminate in three distinct areas, namely, calyces (input area) of the mushroom body and olfactory areas and non-olfactory areas of the lateral protocerebrum, the last area being called the posterior lateral protocerebrum (Frank et al., 2015). The mushroom body is a multisensory integration center that participates in learning and memory. The olfactory area of the lateral protocerebrum is defined as termination areas of olfactory projection neurons. Similarly, in the cockroach, cold projection neurons terminate in the calyces, the olfactory area of the lateral horn and non-olfactory area of the lateral horn, the last area being close to but distinct from termination areas of olfactory projection neurons. Termination areas of warm and warm-cold projection neurons in the cockroach have not been characterized. Transmission of thermosensory signals to different protocerebral regions may be related to the fact that thermosensory signals are used for controlling many forms of thermosensory behaviors. Notably, in the cockroach, termination areas of cold projection neurons in the lateral protocerebrum are highly overlapped with those of moist and dry projection neurons (Nishino et al., 2003). An intimate correspondence between thermosensory processing and hygrosensory processing is further evidenced by the presence of presumed bimodal projection neurons with dendrites in both hygro- and thermo-receptive glomeruli (Nishino et al., 2003).

## Conclusion and perspectives

In conclusion, we suggest that the basic organization of the thermosensory processing system of flies and that of cockroaches are very similar, although they are equipped with different types of thermoreceptors on the antennae. We point out several issues that may be important for further elucidation of the basic principles of thermosensory processing and their roles in the control of thermosensory behavior. First, more effort is needed to investigate whether two-receptor thermosensory system could be found in insects other than flies for better elucidating evolutionary relationship between the two-receptor and one-receptor systems. Blood-sucking bugs *Rhodnius prolix* (Hemiptera) detect infrared to locate remote warm-blooded host, and this is achieved by receptors that respond differently to infrared radiation and warming (Zopf et al., 2014). It would be interesting to see if they have any evolutionary relationship with warm receptors of flies. Second, although recent behavioral studies have focused on elucidation of receptors and neurons participating in rapid avoidance of noxiously high and low temperatures (Gallio et al., 2011; Frank et al., 2015), more attention should be paid to the neural basis of navigation in a shallow temperature gradient in the air. Third, it should be pointed out that orientation behavior toward appropriate temperature areas is similar to that toward odor or water sources, and thus signals from all of these antennal remote-sensing systems may converge to neural circuits that drive similar behavioral programs. Fourth, temperature affects metabolism and affects all physiological functions, and thus temperature signals may be sent to diverse brain regions. Finally, thermosensation and hygrosensation are closely related to each other, as evidenced by perception of humidity being highly influenced by temperature and vice versa in humans (Filingeri et al., 2013; Filingeri, 2015) and cockroaches (Nishino et al., 2003). Integration of thermosensory and hygrosensory signals in the lateral protocerebrum is a fascinating future research subject, and both electrophysiological studies on cockroaches and transgenic approaches in fruit-flies are promising for elucidation of this subject.

Insects are excellent materials for elucidation of basic principles of thermosensory processing due partly to the richness of experimental approaches available. Optogenetics and other powerful genetic tools available for the fruit fly, electrophysiological analysis that is feasible in large insects such as cockroaches, targeted genome-editing technique with CRISPR/Cas9 that is applicable to a variety of insect species, and many other experimental tools such as optophysiology and ablation should provide a solid basis for further investigation. Moreover, diversity and sophistication of insect thermosensory systems, as evidenced by infrared receptors of blood-sucking bugs (discussed above) and those of some beetles that are attracted by forest fires (Kreiss et al., 2007), allow selection of suitable model animals depending on the purpose of research.

## Author contributions

MM, HN, and FY wrote the manuscript and approved the final version.

## Funding

This study was supported by Grants-in-Aid for Scientific Research from the Ministry of Education, Science, Culture, Sports and Technology of Japan (No.24370030 and No. 24657049 to MM and No. 26440175 to HN).

### Conflict of interest statement

The authors declare that the research was conducted in the absence of any commercial or financial relationships that could be construed as a potential conflict of interest.
